# Shear Stress-Triggered Deformation of Microparticles in a Tapered Microchannel

**DOI:** 10.3390/polym13010055

**Published:** 2020-12-25

**Authors:** Cheolheon Park, Junghyun Bae, Yeongjae Choi, Wook Park

**Affiliations:** 1 Department of Electronic Engineering, Kyung Hee University, Deogyeong-daero, Giheung-gu, Yongin-si, Gyeonggi-do 17104, Korea; pakchulhun@khu.ac.kr (C.P.); baejung@khu.ac.kr (J.B.); 2 Nano Systems Institute, Seoul National University, 1, Gwanak-ro, Gwanak-gu, Seoul 08826, Korea; yeongjae@snu.ac.kr; 3 Institute for Wearable Convergence Electronics, Department of Electronic Engineering, Kyung Hee University, Deogyeong-daero, Giheung-gu, Yongin-si, Gyeonggi-do 17104, Korea; 4 Institute for Wearable Convergence Electronics, Department of Electronics and Information Convergence Engineering, Kyung Hee University, Deogyeong-daero, Giheung-gu, Yongin-si, Gyeonggi-do 17104, Korea

**Keywords:** deformation, void fraction, spiral

## Abstract

We demonstrate that it is possible to produce microparticles with high deformability while maintaining a high effective volume. For significant particle deformation, a particle must have a void region. The void fraction of the particle allows its deformation under shear stress. Owing to the importance of the void fraction in particle deformation, we defined an effective volume index (V*) that indicates the ratio of the particle’s total volume to the volumes of the void and material structures. We chose polyethylene glycol diacrylate (Mn ~ 700) for the fabrication of the microparticles and focused on the design of the particles rather than the intrinsic softness of the material (E). We fabricated microparticles with four distinct shapes: discotic, ring, horseshoe, and spiral, with various effective volume indexes. The microparticles were subjected to shear stress as they were pushed through a tapered microfluidic channel to measure their deformability. The deformation ratio R was introduced as R = 1−W_deformed_/D_original_ to compare the deformability of the microparticles. We measured the deformation ratio by increasing the applied pressure. The spiral-shaped microparticles showed a higher deformation ratio (0.901) than those of the other microparticles at the same effective volume index.

## 1. Introduction

Red blood cells (RBCs) move throughout the circulatory system, consisting of tiny capillary networks in some regions. These cells, including mammalian erythrocytes, feature a type of volumetric distortion reminiscent of a cigar when squeezed through very small capillaries [1]. The structure of RBCs is characterized by several unique properties, including their biconcave discoidal shape and mechanical flexibility that have so far been unachieved by synthetic particles [2]. Initially, the diameter of RBCs is larger than that of capillaries; therefore, erythrocytes must change their shape under the applied shear stress as they are pushed through such blood vessels. Consequently, they can move through the narrow capillary network, releasing oxygen as they pass [3]. In this regard, the elastic deformability of RBC membranes has been examined using various methods, including rheoscopy [4], ektacytometry [5], and micropipetting [6], to evaluate their distortion inside a capillary model designed to characterize the sickle-cell disease [7].

These unique properties of RBCs are essential for pharmaceutical delivery carriers. Researchers have created a library of artificial media, such as lipid vesicles [8], emulsions [9], synthetic particles, and microbeads [10], to be used as pharmaceutical delivery carriers with high encapsulation efficiency and high deformability for delivery through the entire circulatory system. Additionally, such deformable and extensively circulating particles may find utility in the field of medical imaging or as targeted drug carriers, where long circulation times and varied biodistributions are often desirable characteristics [11]. However, few geometries simultaneously allow a high deformation and high encapsulation efficiency similar to those demonstrated by RBCs.

To obtain smooth circulation through the capillary system, reducing the carrier’s volume is an alternative method. However, such small (<1 μm in diameter, *d*) carriers (i.e., nanoparticles and lipid vesicles) have extremely limited carrying capacity because their size is proportional to *d*^3^. Maintaining the capacity of a single carrier by introducing morphological flexibility, without reducing the volume, can expand the use of microparticles as a pharmaceutical delivery tool, providing high capacity in a single carrier. Here, we demonstrate that it is possible to produce microparticles on a scale of tens of micrometers that can achieve a high degree of deformability, or deformation ratio (R), while maintaining a high effective volume, expressed by the effective volume index (V*).

Optofluidic maskless lithography (OFML) is a versatile tool for fabricating polymeric microparticles with various shapes, such as cubes or cylinders. This simple fabrication method for multifunctional microparticles, obtained via direct exposure to ultraviolet (UV) light through the lens of a microscope, has allowed the production of color-barcodes [12], anti-counterfeiting fingerprints [13], deployable structures [14], and encoded chemical-laden microparticles [15]. The materials used in the manufacture of these microparticles include PEGDA [9,16], trimethylolpropane triacrylate (TPMTA), poly(methyl methacrylate) (PMMA), and polyurethane acrylate (PUA) [17], all of which are elastic materials that can be responsive to external stress. Interestingly, Chen et al. [18] recently demonstrated the simple distortion of microparticles inside a microfluidic channel, where PUA microparticles showed a type of shear-induced distortion due to their intrinsic elasticity (Young’s modulus, *E* ~ 1000 MPa). In addition, as shown by the recent progress in soft materials research, intrinsic softness helps produce various flexible devices, such as displays, semiconductors, and sensors. Their deformability is dependent on their shape because solid materials feature extrinsic flexural rigidity [17].

Based on these previous studies, we investigated how elastic microparticles may be distorted by external fluidic stress by examining the particle shape’s role. In this study, we chose a single material for the fabrication of microparticles (i.e., PEGDA) to focus on extrinsic designs (i.e., wall thickness and width) rather than on intrinsic softness (*E*) [19]. We constructed a microfluidic channel with a tapered outlet to allow easy generation of microparticles of various shapes, followed by in-situ measurements of their deformability under shear stress. Shear stress is one of the major factors in the construction of stable biofilms is shown in Figures S2.1–2.3 and S3 [20]. Spiral microparticles showed the highest deformability, thereby passing through various narrow outlets. Notably, such high deformability has not been achieved in tests with conventional discotic microparticles. Thus, spiral microparticles are predicted to be suitable as potential drug carriers with easily engineered deformability.

## 2. Materials and Methods

### 2.1. Microfluidic Platforms for the Generation of Microparticles and the In-Situ Measurement of Deformability

For the construction of a microfluidic channel that would serve as the main platform for OFML, a photoresist (SU-8, Microchem Corp., Newton, MA, USA) was purchased and selectively UV-exposed via conventional photolithography. A standard soft-lithography process was then performed, with polydimethylsiloxane (PDMS, Sylgard 184, Dow Corning, Vendor, Seoul, Korea), on the predefined SU-8 channels. The PDMS layer featured a microfluidic channel (10 μm in width and 25 μm in height) with an inlet and an outlet. The PDMS layer and a PDMS-coated glass substrate were surface-modified in an oxygen-plasma chamber (CUTE-1MPR, Femtoscience, Vendor, Yongin, Korea) before integration. The channel’s outlet was designed in a tapered shape with a gradual decrease in the channel width, creating a physical obstacle like that found in the human body’s capillary systems. The tapered angle θ at the outlet was designed (θ = 10°, 50°) to physically confine the microparticles and, in turn, deform them along the outlet under fluidic shear stress. Note that the endpoint for the outlet (~10 μm) was smaller than the microparticles’ diameter (~100 μm) to induce extreme structural distortion by confinement. The fabricated microparticles cannot escape through the outlet if they cannot be sufficiently deformed. The particle diameter was 100 μm, and the wall thickness was 20 μm).

**Figure 1:**
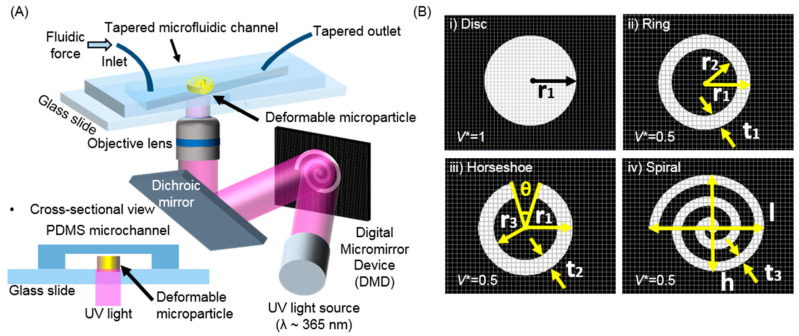
Microfluidic setup for the fabrication and deformation of microparticles with various shapes. (**A**) Schematic illustration of the optofluidic maskless lithography (OFML) setup. (**B**) Digital micromirror device (DMD) images for the selective UV exposure to form the corresponding microparticles. (V* = effective volume index).

**Figure 2:**
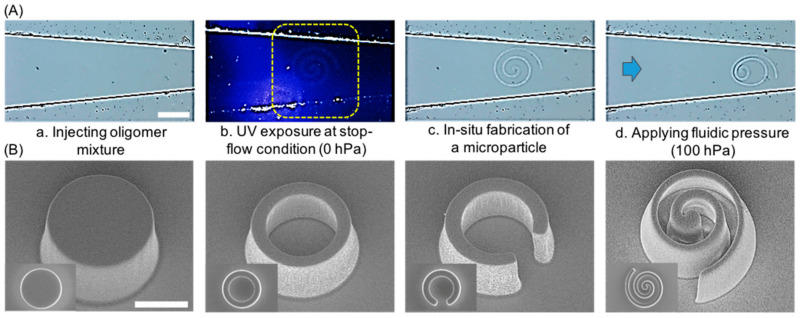
In-situ fabrication of polymeric microparticles in a tapered microfluidic channel for in-situ deformability measurement. (**A**) An in-situ microparticle fabrication process. (scale bar: 100 µm) (**B**) Scanning electron microscopy images of the fabricated microparticles. (scale bar: 75 µm).

After infilling the microchannel with UV-curable PEGDA, the setup was placed onto a microscope for the next OFML procedure. As seen in Figure 1A, the digital micromirror device (DMD, Texas Instruments, Vendor, Seoul, Korea) played a pivotal role in performing spatially guided UV exposure during the direct generation of microparticles, as shown in the scanning electron microscopy images in Figure 2B by designing from the Figure S1.

Such direct inscription of polymeric microparticles allows the design of various shapes, such as discotic, ring, horseshoe, and spiral, in a simple yet direct manner. During UV curing, the permeable PDMS’s oxygen-inhibition layer reduces the friction between the cured PEGDA microparticles and the PDMS channel, thereby allowing the microparticles to slide along the channel easily. The PEGDA resins’ partial curing mainly causes this easy sliding under the oxygen-inhibition layer due to oxygen scavenging. This layer coats the top surface of the PEGDA microparticles with a viscoelastic layer, allowing them to slide smoothly. Figure 1B depicts the simple design of the DMD images that induced various shapes according to the applied mask. A high-intensity UV source (λ ~ 365 nm, LC8, Hamamatsu, 200 W, Vendor, Hamamatsu, Japan) was installed, generating each microparticle in a few hundredths of a millisecond to inscribe the microparticles. In this experiment, the exposure time was 130 ms, and the UV illumination energy was 60 mW/cm^2^. For high pattern fidelity on the microscale, an objective lens with a magnification of 20× was used during the UV exposure. The microparticles have been synthesized after 130 ms of UV irradiation in the microfluidic channel. Under optimized conditions, 100 microparticles can be synthesized in 20 s.

### 2.2. Generation and Observation of the Deformable Polymeric Particles

The role of the extrinsic factor of particle shape in deformability was examined. We chose to use a PEGDA mixture with a 10 vol% fraction of the photoinitiator (Igacure 1173, BASF, Vendor, Seoul, Korea). We fabricated four distinctive microparticle shapes, including a discotic shape (as a control without engineering consideration), as well as ring, horseshoes, and spiral shapes that featured a void region inside their geometry. The void fraction allowed them to be sensitive to external mechanical disturbances, as shown in recent Kirigami strategies with 2D, in-plane structures [21]. A conceptual value of the effective volume index (V*) was defined by dividing the real volume of the particle (*V*_particle_) microparticles and showed a value of V* = 1, while the others were each designed to have V* = 0.5, allowing for easy occupation volume (*V*_total_ = *V*_particle_ + *V*_vacancy_). According to this definition, the discotic deformation reduces their flexural rigidity (Figure 1B).

It can be observed that the intrinsic softness of the PEGDA microparticles allows them to recover their shape after passing through the microchannel outlet. This shape recovery may potentially allow multiple passes of the microparticles through the capillary system and allow them to retain their geometrical advantage for payload delivery at the target site. Quantitative evaluation of shape retention is difficult to consider because the physical properties are slightly different from those at the bulk scale [22,23].

### 2.3. Deformation Measurements and Modeled the Drug-Releasing Ability of Microparticles

The internal fluids were controlled by a microfluidic control system (MFCS^TM^-EZ, Fluigent) under various pressures ranging from 0 to 1000 hPa in 100 hPa increments (Figure 2A) to obtain constant and continuous pressure on each microparticle. Before each UV exposure, the flow was temporarily stopped to prevent the resins’ movement during microparticle fabrication. The microparticles were fabricated by short UV exposure. After fabrication, the dynamic pressure was reapplied to the resin, up to 1000 hPa, to move the microparticles toward the deformation region at the ~10 μm outlet. High-resolution field emission scanning electron microscopy (HR-FESEM, MERLIN, Carl Zeiss, Vendor, Seoul, Korea) was used to observe the topography of the fabricated microparticles with a top view and a tilted view (Figure 2B). For the modeled drug release experiment, the microparticles were immersed in anhydrous ethyl alcohol (99%, Daejung, Seoul, Korea) with rhodamine (Polyscience), which passively diffused from the microparticles over time. After drying thoroughly, the microparticles were placed onto agarose sheets, allowing passive diffusion of the rhodamine on the sheet over a fixed release time of 10 min. Finally, the microparticles were removed from the agarose sheets for observation via fluorescence microscopy (IX71, Olympus, Vendor, Seoul, Korea).

## 3. Results and Discussion

**Figure 3:**
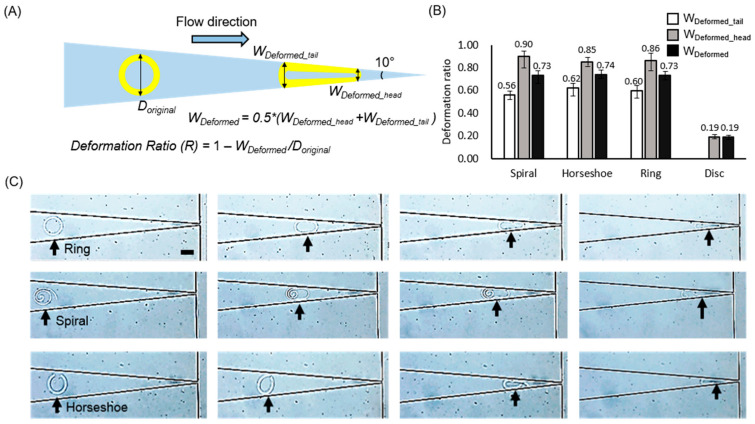
In-situ measurements of the deformability of microparticles. (**A**) Schematic illustration of the deformation ratio (*R* = 1−W_deformed_/D_original_). D_original_: diameter of the microparticle, W_deformed_: average width at the head (W_deformed_head_) and tail (W_deformed_tail_) of the microparticle, where the tapered angle at the outlet is 10°. (**B**) Measured deformation ratio of four designs: spiral, horseshoe, and ring (V* = 0.5), and discotic microparticles (V* = 1). The error bars represent the standard deviations (*n* = 10). (**C**) Optical images of each microparticle to show their deformability in the microchannel. (scale bar: 100 µm).

**Figure 4:**
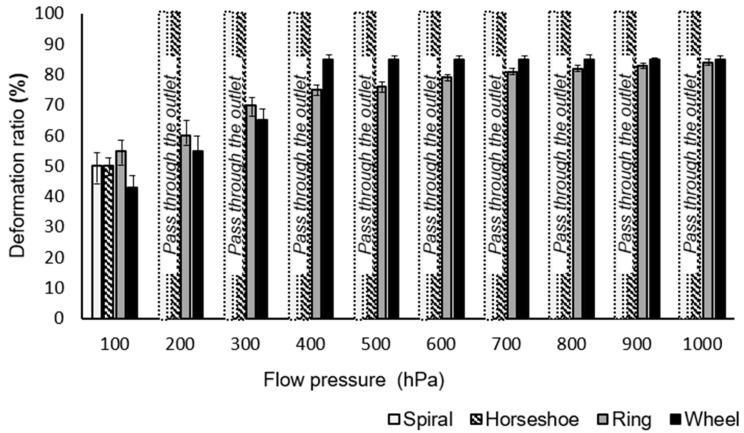
Deformation ratios for three types of microparticles with the same effective volume index (V* = 0.5), with respect to flow pressure. (0–1000 hPa). In particular, the spiral and horseshoe microparticles can be shown to pass through the channel outlet under a flow pressure of 200 hPa or greater. The error bars represent the standard deviations (*n* = 10).

The schematic in Figure 3A presents an example of the measurement of the deformability of the microparticles, showing the elliptical shape of a ring microparticle after distortion. The structural distortion was caused mainly by fluidic shear stress, created by in-plane confinement along the tapered channel. This distortion may differ from an RBC deformation in a 3D circular capillary since the deformation of the immersed structures is strongly dependent on the hydrodynamic stresses [24]. Accurate modeling of a tube-shaped blood vessel or capillary is under consideration. These experiments are the first step toward demonstrating the feasibility of designing this type of carrier on a microscale. The deformation ratio *R* was introduced as *R* = 1−W_deformed_/D_original_, where D_original_ is the diameter of the microparticle without any deformation and W_deformed_ is the average of the width at the head and the tail of the microparticle (W_deformed_head_ and W_deformed_tail_, respectively) to compare the deformability of each microparticle. We measured W_deformed_head_ and W_deformed_tail_ across the microparticle when the microparticle stopped moving under the given pressure (100 hPa) (Figure 3B). To measure the deformation ratios with respect to the flow pressure, we used a microfluidic channel with a 50° slope. The flow pressure in the channel ranged from 0 to 1000 hPa and was controlled using an MFCS device such that the pressure increased by steps of 100 hPa. The four types of microparticles (spiral, horseshoe, ring, and discotic), each with the same outer diameter and effective volume index (V* = 0.5), were tested in the microfluidic channel. Figure 4 shows the deformation ratios for these three microparticles.

The non-submissive discotic microparticles showed the lowest deformability *R* ~ 0.09, suggesting a decrement in diameter of less than 10% against shear stress. In these experiments, the discotic microparticles had an initial diameter of 150 μm and stopped in the channel with only a slight decrease to ~121.5 μm in diameter. This is because, in part, microparticles without a void fraction cannot significantly deform owing to their inherent rigidity. Thus, conventional particle systems that contain no void fractions may not be as effective as deformable carriers.

In contrast to the discotic microparticles, the other three geometries showed greater structural deformation when the same pressure (~100 hPa) was applied to each sample. With the height and thickness of each of the microparticle walls at 25 and 20 μm, respectively, none were able to pass through the ~10 μm outlet without deformation. However, reducing the thickness of the microparticle walls from 20 to 10 μm allowed in-plane rotation of both the spiral and horseshoe microparticles, resulting in their escape through the outlet with the same applied pressure as with the 20 μm wall thickness. The ring microparticles did not escape through the outlet, even with a reduced wall thickness.

Even though the ring microparticle has a void fraction giving it the same effective volume index as the spiral and horseshoe microparticles, if the thickness of its wall is larger than half of the width of the microchannel outlet, it cannot pass through the outlet. Near the outlet, the microparticle walls meet each other, making the width of the arc at the head of the microparticle approximately twice the width of the microchannel outlet. The deformed microparticles cannot pass through the outlet with a narrow width due to the outer thickness of the structure (Figure S2.1). Because the ring microparticles do not have an open curve, they cannot open to allow only a single wall to pass through the outlet, as is possible with the spiral and horseshoe-shaped microparticles.

The spiral microparticles showed the largest difference in *R* (∆*R* ~ 0.344) between the widths of their head and tail arcs (W_deformed_head_ and W_deformed_tail_). This difference occurs because of in-plane rotation and the nanoparticles’ corresponding orientation under confinement. Specifically, the spiral microparticle consisted of a self-similar open loop in its geometry, yielding the easiest deformation against shear stress among all the designs in this study. A strand-like form occurs upon unfolding the spiral microparticles (Supplementary Figure S1), a type of deformation not presented by the other shapes in this study.

The discotic and ring microparticles could not pass through the end of the tapered channel, even under the maximum pressure (1000 hPa) because the structures do not have an open curved shape. The spiral and horseshoe microparticles passed through the end of the tapered channel under low pressure (~200 hPa) because of their open curved shape. This observation demonstrates that, even if the microparticles have the same effective volume index, they may have different relative thicknesses, depending on their design. In this case, the microparticles with an open curved structure can be capable of a more complex type of deformability than microparticles with a closed curved structure, even though they have the same effective volume index. We also qualitatively observed that all the particles recovered their original shape after passing through the outlet due to their intrinsic softness.

In this study, the unfolded structures were not recovered for quantitative assessment after they escaped via the outlet. Such an assessment requires consideration of the elastic properties of the constituent material. This consideration is not possible in this study, as we chose a single material for fabrication. However, shape recovery and microparticle durability are important fabrication parameters to consider in further studies of the potential use of these microparticles as deformable drug carriers. In particular, the spiral shape seems to be most suitable for microcarriers because of its outstanding ability to pass through the modeled capillary. We found that microparticles fabricated in a particular condition, such as low-intensity UV light, show a higher possibility of not returning to their original shape after unfolding. Future research should analyze deformable microparticles considering design parameters and material parameters.

**Figure 5:**
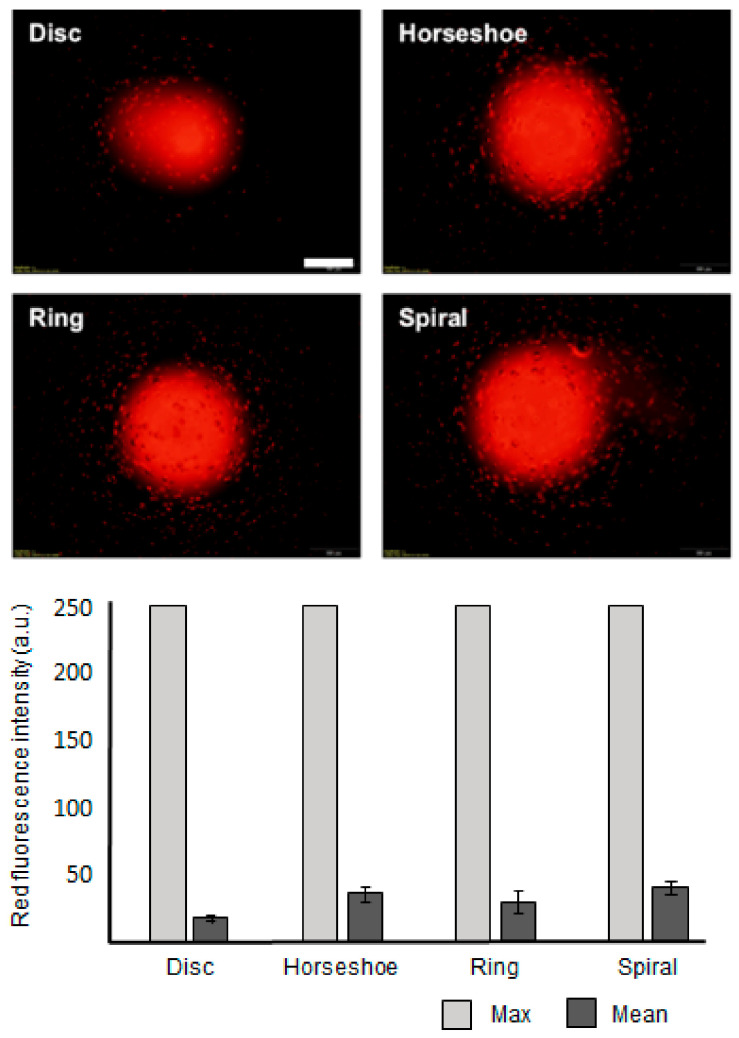
Pharmaceutical releasing ability of the discotic, horseshoe, ring, and spiral microparticles. Rhodamine was selected as the modeled pharmaceutical. Inset images show the diffusion of rhodamine onto agarose sheets after 10 min. The error bars represent the standard deviations (*n* = 10) (scale bar: 500 µm).

Figure 5 shows the pharmaceutical releasing ability of the four microparticle shapes presented thus far. RBCs release oxygen as they move through the capillary system. Similarly, PEGDA microparticles could deliver their content via diffusion. The four microparticle shapes with the same effective volume index and heights were examined to compare their potential diffusion-based drug release ability. We placed the microparticles containing rhodamine onto an agarose sheet and allowed diffusion to proceed for 10 min. The inset images in Figure 5 show the diffused rhodamine, visualized via fluorescent microscopy, displaying a clear difference in the amounts released by the four microparticle shapes. From the data, the spiral microparticles diffused the rhodamine to the greatest extent (Table S2). The graph indicates that a larger surface area, including the void fraction of the horseshoe, ring, and spiral microparticles, promotes greater diffusion. In these experiments, we employed 2D agarose sheets as the pharmaceutical receptor. Diffusion from spiral microparticles may be more effective when they are surrounded by liquid media or blood.

The spiral-shaped microparticles were proven to be suitable as a deformable carrier to deliver substances through a very small capillary system owing to their high deformability and substantial drug release ability. (Figure 5 and Figure S4) However, to use these deformable carriers in the human capillary system, the microparticle’s dimensions must be reduced to tens of micrometers in diameter and less than a micrometer in wall thickness. Additionally, in the in-situ deformability measurement system, we applied pressure to the microcarrier with an oligomer mixture rather than a biological buffer solution. After the microcarrier’s in-situ fabrication, it is not feasible to exchange the oligomer mixture with a biological buffer in the microfluidic channel because the fabricated microparticle may be washed out during the fluid exchange in our current system. Our system will be modified for further study to allow biological aqueous buffers such as saline or a dextrose solution. We do not anticipate the PEGDA microparticles’ instability in these solutions, but this must be demonstrated with the designed microcarrier dimensions appropriate for the target channel diameter. In addition, the deformation index of RBCs is 0.8–1 [25]. Applying this measurement to our calculation in Figure 3, the deformation ratio of RBCs is approximately 80%. In our investigation, we achieved deformation ratios higher than 80%, except for the disk particles (19%). We believe that by increasing deformability to more than four folds, we can extend the application areas of macroparticles to, e.g., drug delivery.

## 4. Conclusions

In conclusion, we have presented a simple yet robust method for the in-situ fabrication and in-situ deformability measurement of structured hydrogel microparticles based on the extrinsic design rather than on the intrinsic softness using the OFML fabrication method. We compared several designs for deformable microparticles with the same effective volume ratio. The spiral-shaped microparticles were demonstrated to be highly deformable under fluidic shear stress in a microfluidic channel, showing that it may be feasible as a carrier owing to its large surface area. We have shown that, under shear stress, the fabricated spiral shapes can rotate and unwind to pass through an outlet with a diameter 90% smaller than the outer diameter of the spiral without fractures or cracks in the microparticles. We investigated the drug release ability using fluorescent molecules to visualize the differences in diffusion between the disc and spiral-shaped microparticles. We have shown that the deformable spiral design for microparticle systems, with further size reduction, can potentially serve as a new type of drug carrier for use in human life sciences.

## Data Availability

Data available in a publicly accessible repository.

## Supplementary Materials

The following are available online at https://www.mdpi.com/2073-4360/13/1/55/s1. Table S1: Expressions for calculating the parameters of the particles. Figure S1: Monitoring the escape of the spiral particles. Note that the spiral particle undergoes in-plane rotation, thereby escaping the tiny outlet. (scale bar: 100µm). Figure S2.1: Deformation ratios of the ring particle by increasing flow pressures, (A) Deformation ratio at 100 hPa, (B) 200 hPa, (C) 300 hPa, (D) 400 hPa, (E) 500 hPa, (F) 600 hPa, (G) 700 hPa, (H) 800 hPa, (I) 900 hPa, (J) 1000 hPa. (Scale bar: 50 µm). Figure S2.2. Deformation ratios of the wheel particle by increasing flow pressures, (A) Deformation ratio at 100 hPa, (B) 200 hPa, (C) 300 hPa, (D) 400 hPa, (E) 500 hPa, (F) 600 hPa, (G) 700 hPa, (H) 800 ~ 1000 hPa. (Scale bar: 50µm). Figure S2.3. Deformation ratios of the horseshoe and spiral particles by increasing flow pressures, (A) Deformation of horseshoe particle at 100 ~ 200 hPa, (B) Deformation of spiral particle at 100 ~ 200 hPa. (Scale bar: 50µm). Figure S3: Recovery of the spiral particle shape after passing through the tapered channel at flow of 200 hPa (Scale bar: 50 µm). Figure S4: Measurement of the diffusion area of the Rhodamine onto the agarose sheet. The area for the spiral particle is 112% larger than the area for the discotic particle, although the particles have the same effective volume index. (*V** = 0.5), as shown in Supplementary Table S2. Measurement of the red fluorescence intensity of the diffused Rhodamine-B on the agarose sheet. The spiral particle is 109% greater in intensity than the discotic particle, although the particles have same effective volume index. (*V** = 0.5).
