# Supplementary material for: Shear Stress-Triggered Deformation of Microparticles in a Tapered Microchannel
Source: Polymers (Basel). 2020 Dec 25;13(1):55. doi: 10.3390/polym13010055 (PMC7795621; doi:10.3390/polym13010055)
Supplement: Supplementary file 1 [file polymers-13-00055-s001.pdf]

polymers

MDPI

Supplementary Materials

Shear Stress Triggered Deformation of Microparticles in a Tapered Microchannel

Cheolhen Park [1], Junghyun Bae [1], Yeongjae Choi [2] and Wook Park [1,3,4,*]

1 Department of Electronic Engineering, Kyung Hee University, Deogyeong-daero, Giheung-gu, Yongin-si, Gyeonggi-do 17104, Korea; pakchulhun@khu.ac.kr (C.P.); baejung@khu.ac.kr (J.B.)
2 Nano Systems Institute, Seoul National University, 1, Gwanak-ro, Gwanak-gu, Seoul 08826, Korea; yeongjae@snu.ac.kr (Y.C.)
3 Institute for Wearable Convergence Electronics, Department of Electronic Engineering, Kyung Hee University, Deongyeong-daero, Giheung-gu, Yongin-si, Gyeonggi-do 17104, Korea
4 Institute for Wearable Convergence Electronics, Department of Electronics and Information Convergence Engineering, Kyung Hee University, Deongyeong-daero, Giheung-gu, Yongin-si, Gyeonggi-do 17104, Korea
* Correspondence: parkwook@khu.ac.kr; Tel.: +82-31-201-3465

Table S1. Expressions for calculating the parameters of the particles.

$\pi r_1^2 \times V^* = \pi r_1^2 - \pi r_2^2$	(1.1)
$r_1 - r_2 = t_1$	(1.2)
$\pi r_1^2 \times V^* = \pi r_1^2 - \pi r_3^2 - (\pi r_1^2 - \pi r_3^2)\frac{\theta}{360^\circ}$	(1.3)
$r_1 - r_3 = t_2$	(1.4)
$h/2 \times l/2 \times \pi \times V^* = Length of spiral pattern \times t_3$	(1.5)

The various parameters of these patterns are calculated by using the expressions in Table S1. 2~6. Because we produce the particles in a microfluidic channel, the upper surface of the particles is considered when the particles are designed. For example, Table 1.2 shows the expression for the ring pattern, Table 1.4 is the expression for the horseshoe pattern, and Table 1.6 is the expression for the spiral pattern. The spiral pattern is designed in MATLAB to control the length of the pattern and the thickness of the line.

For example, when we design the spiral particle (thickness of the particle = 50 μm, h = 400 μm, l = 600 um and V^* = 0.5).

The parameters of the discotic and ring particles are:
Diameter of discotic particle = 346 μm;
Thickness of ring particle = 51 μm.

Further, we have to decide the θ for the horseshoe particle (in this case, θ = 30°).

Thickness of horseshoe particle = 67 μm.

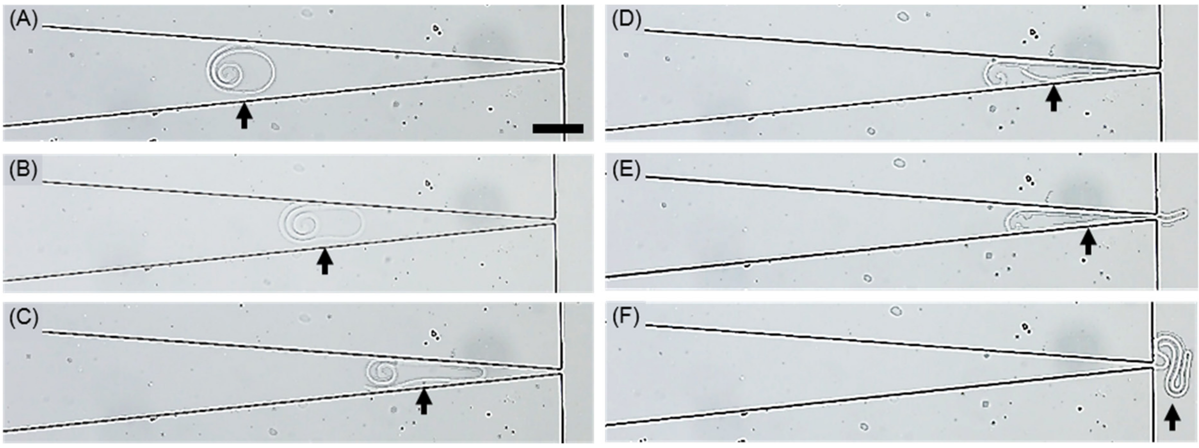

Figure S1. Monitoring the escape of the spiral particles. Note that the spiral particle undergoes in-plane rotation, thereby escaping the tiny outlet. (scale bar: 100 μm).

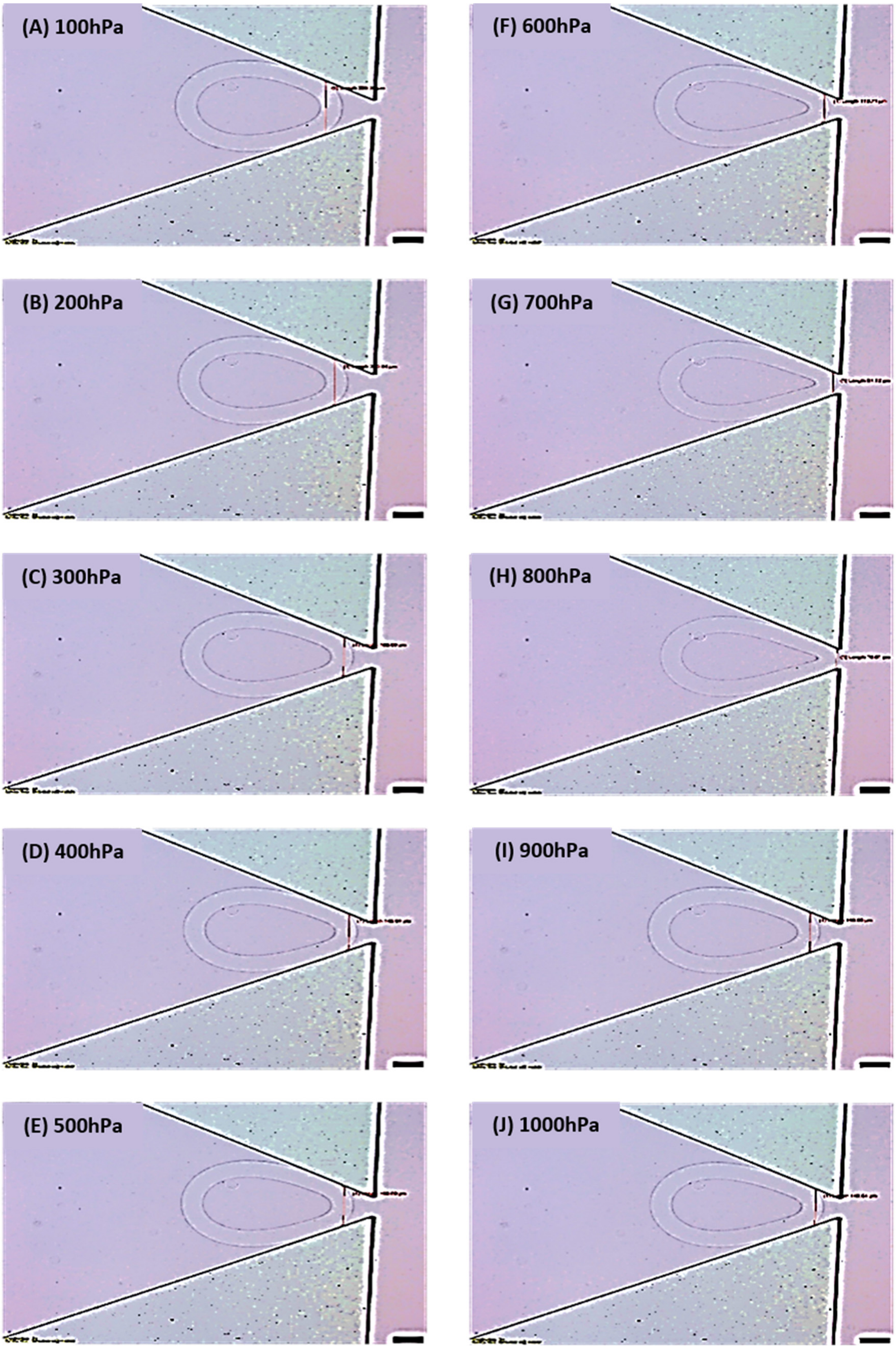

Figure S2.1. Deformation ratios of the ring particle by increasing flow pressures, (**A**) Deformation ratio at 100 hPa, (**B**) 200 hPa, (**C**) 300 hPa, (**D**) 400 hPa, (**E**) 500 hPa, (**F**) 600 hPa, (**G**) 700 hPa, (**H**) 800 hPa, (**I**) 900 hPa, (**J**) 1000 hPa. (Scale bar: 50 μm).

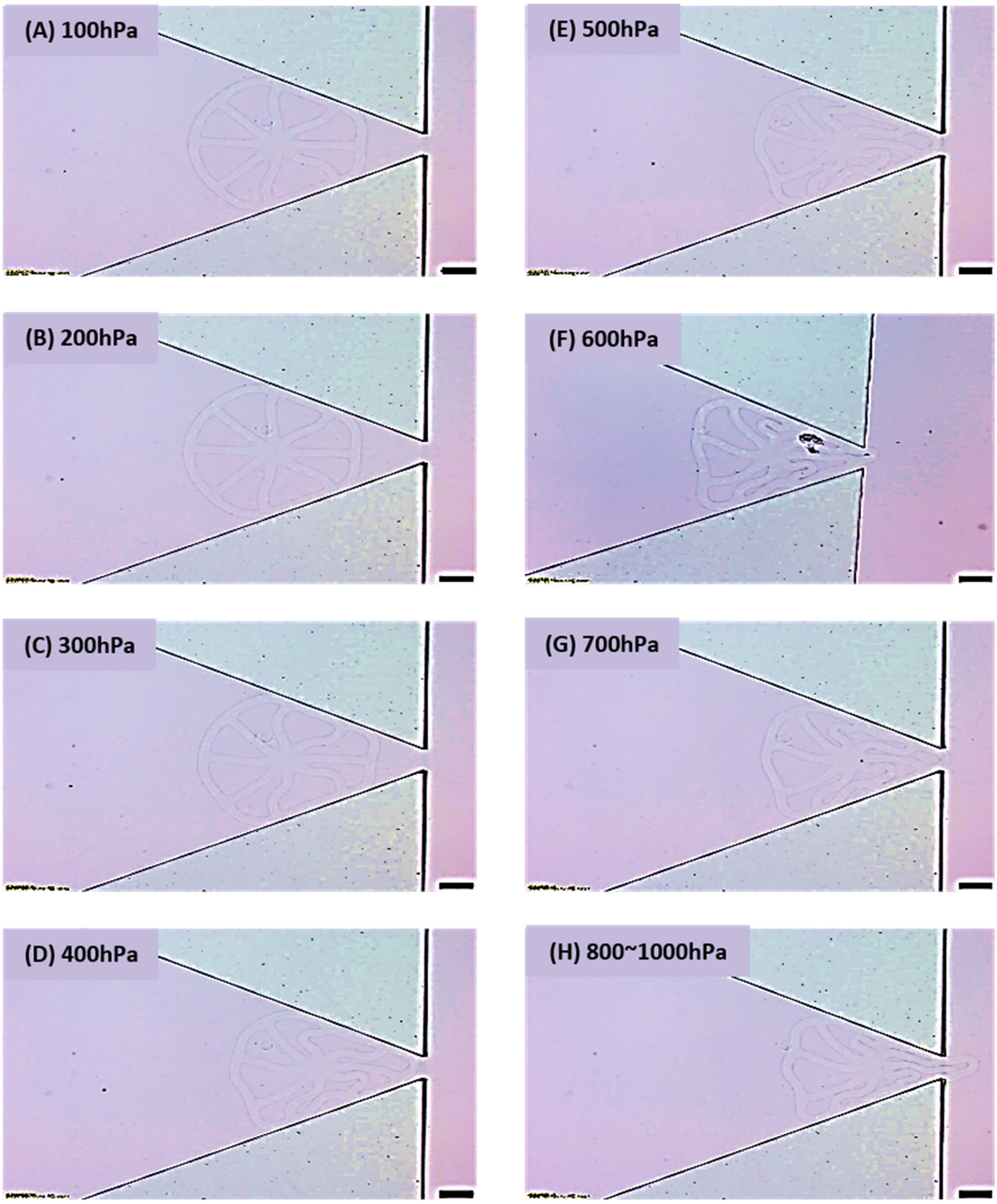

Figure S2.2. Deformation ratios of the wheel particle by increasing flow pressures, (**A**) Deformation ratio at 100 hPa, (**B**) 200 hPa, (**C**) 300 hPa, (**D**) 400 hPa, (**E**) 500 hPa, (**F**) 600 hPa, (**G**) 700 hPa, (**H**) 800–1000 hPa. (Scale bar: 50 μm).

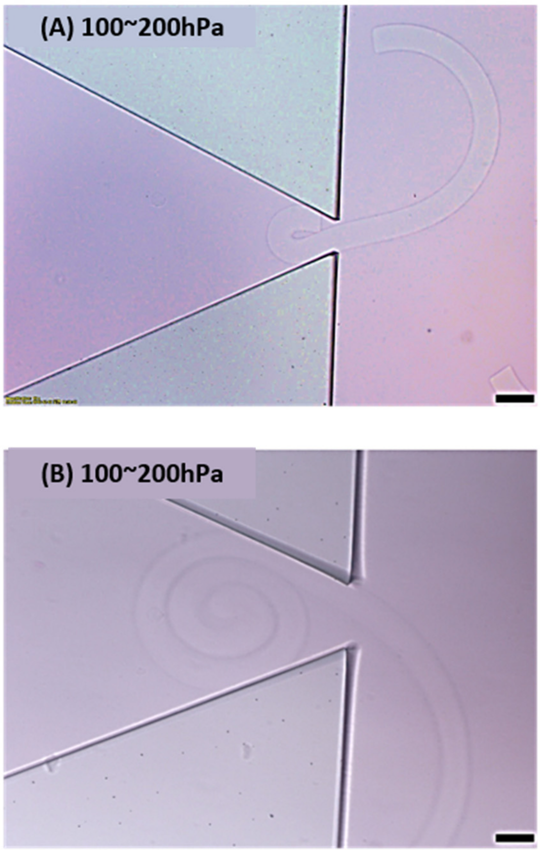

Figure S2.3. Deformation ratios of the horseshoe and spiral particles by increasing flow pressures, (**A**) Deformation of horseshoe particle at 100–200 hPa, (**B**) Deformation of spiral particle at 100–200 hPa. (Scale bar: 50 μm).

Even at the maximum flow pressure (1000 hPa), the ring and wheel particles can't escape from the channel because they do not have an open curve to allow greater deformation (Figure S2.1.–2.2.). On the other hand, the horseshoe and spiral particles have an open loop that allows the particles to sufficiently deform to escape from the channel at the flow pressure of 200 mph (Figure S2.3.).

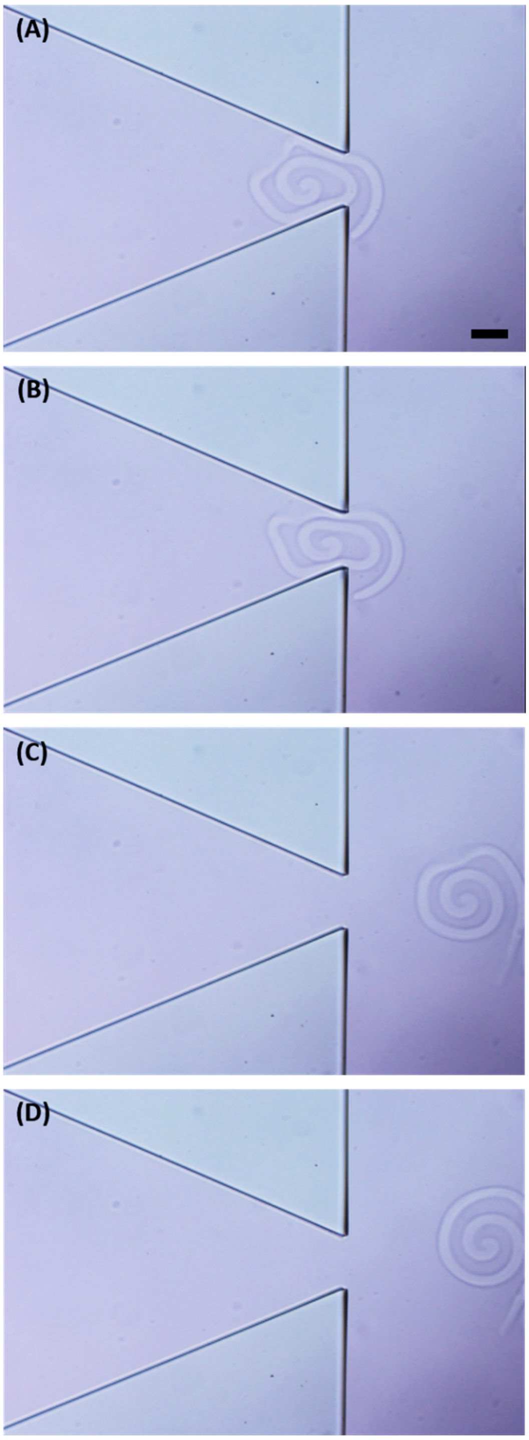

Figure S3. Recovery of the spiral particle shape after passing through the tapered channel at flow of 200 hPa (Scale bar: 50 μm).

The spiral particle recovered its original structure after deformation and passing through the channel. (Because we focused on deformation of the particle at the channel outlet, the right side in the images is less clear.)

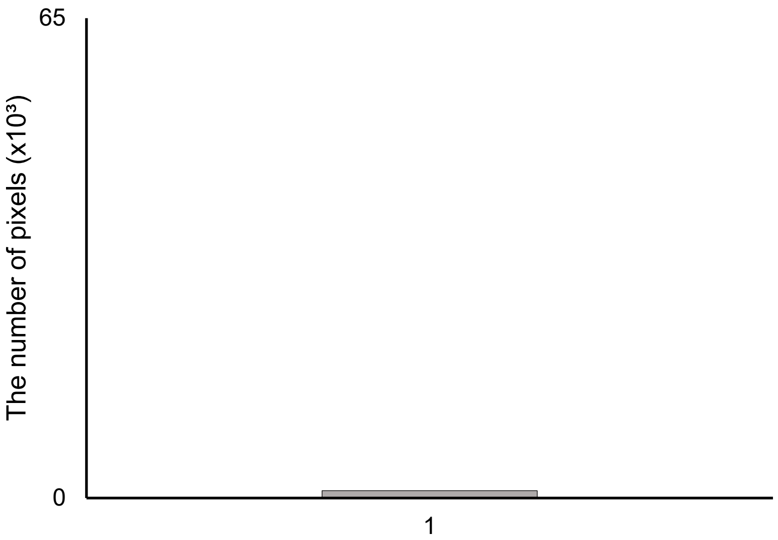

Figure S4. Measurement of the diffusion area of the Rhodamine onto the agarose sheet. The area for the spiral particle is 112% larger than the area for the discotic particle, although the particles have the same effective volume index. (V^* = 0.5).

Table S2. Measurement of the red fluorescence intensity of the diffused Rhodamine-B on the agarose sheet. The spiral particle is 109% greater in intensity than the discotic particle, although the particles have same effective volume index. (V^* = 0.5).

	**Max**	**Mean**	**StdDev**
Disc	255	19.246	48.775
Horseshoe	255	37.638	66.376
Ring	255	29.754	62.12
Spiral	255	40.401	62.23

Following the diffusion of the Rhodamine from the discotic, horseshoe, ring, and spiral particles onto the agarose sheet, the degree of diffusion was measured by the fluorescence intensities (Figure 5). Additionally, Figure S4 shows the diffused area of the Rhodamine-B. The areas were measured by ImageJ Tool.
